# Independent Predictors of Mortality in Torso Trauma Injuries

**DOI:** 10.3390/jcm9103202

**Published:** 2020-10-03

**Authors:** Roberto Bini, Caterina Accardo, Stefano Granieri, Fabrizio Sammartano, Stefania Cimbanassi, Federica Renzi, Francesca Bindi, Laura Briani, Osvaldo Chiara

**Affiliations:** 1 Trauma Center and Metropolitan Trauma Network Department, Niguarda Hospital, 20121 Milan, Italy; roberto.bini@ospedaleniguarda.it (R.B.); fabrizio.sammartano@ospedaleniguarda.it (F.S.); stefania.cimbanassi@ospedaleniguarda.it (S.C.); federica.renzi@ospedaleniguarda.it (F.R.); francesca.bindi@ospedaleniguarda.it (F.B.); laura.briani@ospedaleniguarda.it (L.B.); 2 Ospedale Maggiore Policlinico, General and Liver Transplant Surgery Unit-Fondazione IRCCS Ca’ Granda, University of Milan, 20122 Milan, Italy; 3 General Surgery Unit, ASST-Vimercate, 20871 Vimercate, Italy; stefano.granieri@asst-vimercate.it; 4 Department of Medical-Surgical Physiopathology and Transplantation, University of Milan, 20122 Milan Italy; osvaldo.chiara@unimi.it

**Keywords:** trauma surgery, hemorrhage, critical care, trauma epidemiology

## Abstract

Noncompressible torso injuries (NCTIs) represent a trauma-related condition with high lethality. This study’s aim was to identify potential prediction factors of mortality in this group of trauma patients at a Level 1 trauma center in Italy. Materials and Methods: A total of 777 patients who had sustained a noncompressible torso injury (NCTI) and were admitted to the Niguarda Trauma Center in Milan from 2010 to 2019 were included. Of these, 166 patients with a systolic blood pressure (SBP) <90 mmHg were considered to have a noncompressible torso hemorrhage (NCTH). Demographic data, mechanism of trauma, pre-hospital and in-hospital clinical conditions, diagnostic/therapeutic procedures, and survival outcome were retrospectively recorded. Results: Among the 777 patients, 69% were male and 90.2% sustained a blunt trauma with a median age of 43 years. The comparison between survivors and non-survivors pointed out a significantly lower pre-hospital Glasgow coma scale (GCS) and SBP (*p* < 0.001) in the latter group. The multivariate backward regression model identified age, pre-hospital GCS and injury severity score (ISS) (*p* < 0.001), pre-hospital SBP (*p* = 0.03), emergency department SBP (*p* = 0.039), performance of torso contrast enhanced computed tomography (CeCT) (*p* = 0.029), and base excess (BE) (*p* = 0.008) as independent predictors of mortality. Conclusions: Torso trauma patients who were hemodynamically unstable in both pre- and in-hospital phases with impaired GCS and BE had a greater risk of death. The detection of independent predictors of mortality allows for the timely identification of a subgroup of patients whose chances of survival are reduced.

## 1. Introduction

NCTIs are particularly frequent in trauma patients. In this scenario, NCTH is a trauma-related condition with an elevated risk of death. Hemorrhage accounts for 40% of civilian trauma-related deaths and more than 90% of military deaths from potentially non-lethal injuries [1]. Although NCTH has been widely explored in battlefield scenarios, little is known about it in civilian urban areas. Epidemiological data from Europe are lacking.

In trauma patients, most early deaths are caused by hemorrhage and occur at a median of 2.6 h after admission [2]. While extremities injuries can be managed with both hemostatic gauzes and tourniquets, NCTHs need to be managed in operating or interventional rooms. The latter requires rapid identification of the source of bleeding and its timely treatment in a context suitable for the emergency.

Trauma centers and trauma networks must be prepared for such kinds of patients. Accordingly, we wanted to explore the magnitude of the phenomenon in our country.

This study aimed to characterize the epidemiology ofNCTIs and NCTH at a Level 1 trauma center in Italy and to identify potential predictors of mortality in this group of trauma patients.

## 2. Materials and Methods

All details about trauma patients managed at our level 1 Trauma center are collected in the Niguarda trauma registry, in which demographic data, mechanism of trauma, pre-hospital and in-hospital clinical conditions, diagnostic/therapeutic procedures, and survival outcome are recorded. The registry is held by a trauma team consultant who is meant to keep it constantly updated, and it is annually revised by the head of the department. All blunt and penetrating trauma patients sustaining NCTI consecutively admitted to Niguarda Trauma Center from 2010 to 2019 were selected from the registry. Several facets need to be considered when defining NCTH: anatomic injury pattern, physiology, and the clinical need for intervention.

NCTI was defined by at least one of the following anatomic criteria: (1) pulmonary injury (massive hemothorax, pulmonary vascular injury) (2) named axial torso vessel injury, (3) ≥ grade 4 solid organ injury according to the abbreviated injury scale classification (AIS, 1998 version [3]), and (4) pelvic fracture with ring disruption.

NCTH was defined as a NCTI in which in addition to the anatomical criteria there was a SBP of <90 mmHg (physiological criteria) and/or the need for immediate hemorrhage control [1,4]

Patients aged < 18 years were excluded from the study.

At our institution, the treatment strategy for hemodynamically unstable patients involves the immediate activation of the massive transfusion protocol along with a prompt diagnosis of hemorrhage through extended focus assessment sonography for trauma (E-FAST) and chest and pelvis X-rays. External sources of bleeding are directly controlled. As suggested by recent evidence, we prefer transfusion of blood products rather than fluids infusion. Patients are deemed eligible for a second level diagnostic with CeCT only if they are hemodynamically stable/responders to transfusion of blood products or if there are signs of increased intracranial pressure (ICP)/neurogenic shock. The decision and timing of surgical/angiographic control of the bleeding is based on the number of blood products transfused (even though a strict threshold is not well established in the literature), the anatomical site of injuries, and hemodynamic response to treatments.

Demographic data, trauma mechanism, vitals, and GCS on the scene and at admission, AIS score of head, chest, abdomen, and extremities, American Society of Anesthesiologists (ASA) score, injury severity score (ISS), revised trauma score (RTS), gas analysis parameters, number of packed red blood cells (PRBCs), fresh frozen plasma (FFP), platelet pools (PP) transfused within the first 24 h, probability of survival (PS) obtained by the Trauma and Injury Severity Score (TRISS) system, length of hospitalization, and survival outcome were retrospectively analyzed.

Data were recorded in a computerized spreadsheet (Microsoft Excel 2016; Microsoft Corporation, Redmond; WA, USA) and analyzed with statistical software (IBM Corp. Released 2017, IBM SPSS Statistics for Windows, Version 25.0. Armonk, NY, USA).

The distribution of each continuous variable was evaluated with Kolmogorov–Smirnov and Shapiro–Wilk tests. Continuous variables were compared by independent sample Mann–Whitney test or independent samples *t*-test where appropriate; categorical variables were analyzed using Pearson’s Chi-squared test or Fischer’s test.

In order to identify independent predictors of mortality, significant variables at univariate analysis (*p* < 0.05) were tested with univariate logistic regression and underwent multicollinearity diagnostics. This intermediate step was introduced to select the variables to be included in a stepwise backward multivariate logistic regression model providing odds ratio and 95% confidence intervals. The Nagelkerke R square was evaluated to assess the proportion of the variance predicted by the model and the Hosmer–Lemeshow test was used to evaluate the goodness of fit.

Patients sustaining pre-hospital or on arrival cardiac arrest were excluded from multivariate analysis due to the remarkable confounding effect of this variable on the outcome.

A *p* value < 0.05 was considered statistically significant.

The institution of a trauma registry for all major traumas admitted to our trauma center has been approved by the Niguarda Ethical Committee, Milan area 3 (record number 534-102018). Given the retrospective nature of the study, a specific ethical review board approval was not required.

## 3. Results

During the study period, 777 patients sustaining blunt and penetrating trauma with NCTI were consecutively admitted to our institution. The median age was 43 years **interquartile range** (IQR) of 30–59; 69% of patients were male and 31% were female. Seven hundred one patients (90.2%) sustained blunt trauma whereas 76 (9.8%) were admitted because of gunshots or stab wounds.

The comparison between survivors and non-survivors pointed out a significantly lower pre-hospital GCS (*p* < 0.001) and SBP (*p* < 0.001) in the latter group. Moreover, expired patients were older (*p* < 0.001), with a greater proportion of American Society of Anesthesiologists: physical status (ASA-PS) >1 (*p* = 0.001) and had an increased incidence of severe head (*p* < 0.001) and chest (*p* = 0.021) injuries. More detailed results are reported in Table 1.

Deceased patients reported a significantly lower GCS (*p* < 0.001) and SBP (*p* < 0.001) even on admission to the emergency department and had a higher median ISS (*p* < 0.001). Furthermore, non-survivors underwent more damage control laparotomies (*p* = 0.014), emergency department thoracotomies (*p* < 0.001), and extraperitoneal pelvic packing (*p* < 0.001). Additional data are reported in Table 2.

The multivariate backward regression model identified age (*p* < 0.001; OR: 1.064; 95% CI: 1.045–1.083), pre-hospital GCS (*p* < 0.001; OR: 0.831; 95% CI: 0.771–0.896), pre-hospital SBP (*p* = 0.03; OR: 0.989; 95% CI: 0.98–0.999), emergency department SBP (*p* = 0,039; OR: 0.989; 95% CI: 0.979–0.999), performance of torso CeCT scan (*p* = 0.029; OR: 0.270; 95% CI: 0.083–0.874), base excess (*p* = 0.008; OR: 0.931; 95% CI: 0.883–0.982), and ISS (*p* < 0.001; OR: 1.05; 95% CI: 1.026–1.075) as independent predictors of mortality (Table 3).

The Hosmer–Lemeshow test highlighted an adequate goodness-of-fit of the model (*p* = 0.212). The model correctly classified 90.1% of cases, and its performance was tested using Nagelkerke R^2^ with a 56.4% predicted variance.

### Analysis of Non-Survivors

Deceased patients accounted for 16.3% of all NCTI patients. Of these, 81.8% (104/127) experienced an early death (in the first 24 h after trauma) while the other 23 patients died of complications such as sepsis or progression of underlying diseases.

In the early death group, the majority of cases (60/104–57.6%) were real NCTH, whereas 35/104 (33.65%) died from NCTI associated with irreversible traumatic brain injury (TBI) with AIS > 3. For the NCTH group, more than 70% of patients sustained multiple organ injuries (at least two different sites of injury).

Of all NCTH patients, 17.8% sustained cardiac arrest during the pre-hospital phase or on arrival in the emergency department. As mentioned in the methods section, these patients were excluded from the analysis of independent predictors of mortality.

## 4. Discussion

We performed a retrospective study of patients suffering from NCTIs and NCTHs who were managed at the Niguarda Trauma Center from 2010 and 2019. In our study, the NCTI patients were mostly males (69%), with a median age of 43 years, who sustained blunt trauma (90.2%). Non-survivors are male with a median age of 57 years and suffered from blunt trauma in 92.3% of cases.

In our center, during the study period, we observed 777 patients, of whom 166 had hemodynamic instability (NCTH). The mortality rate among NCTH patients was 41.5%, whereas among NCTI patients it was 9.4%. Our findings are consistent with those reported in the literature [4].

Focusing on the anatomic district of injury in NCTH patients, our analysis failed to show a prevalence of one over the others. In more than 70% of cases, death was caused by pelvic and chest bleeding simultaneously. Indeed, the involvement of a single anatomic district is rare in NCTI and NTCH patients.

In our study, multivariable analysis identified age, pre-hospital GCS, pre-hospital SBP, emergency department SBP, the performance of torso CeCT scan, base excess, and ISS as independent predictors of mortality.

Age was the only demographic variable identified as an independent predictor of mortality in our analysis, with a 6.4% raise in risk of death per unitary increase. Advanced age is well recognized as an important contributor to increased mortality risk among trauma patients [5]. Older patients have been demonstrated to have significantly greater mortality risk than younger patients with otherwise similar injury severity score (ISS) levels [6].

Prehospital parameters, such as GCS and SBP, had a significant role in predicting mortality. Alarhayem et al. demonstrated that patients with prehospital SBP < 90 mmHg had a two-fold increase in the risk of death compared to their counterparts with SBP above 90 mmHg [7]. Along with hypotension at arrival in the emergency department (ED), our findings describe a patient poorly perfused with ongoing bleeding. A recent manuscript published by Spaite et al. [8] reported an 18% increase in the risk of death for every 10 mmHg drop in SBP when it falls below 120 mmHg [9]. Interestingly, in our study, for every 10 mmHg gain in SPB, we registered an 11% reduction in risk of death.

Low GCS on arrival, high international normalized ratio (INR), and the need for intubation were all found to be risk factors of in-hospital mortality even though multivariate analysis failed to identify them as independent predictors of death [10,11,12].

Base excess and ISS are well-known predictors of death in trauma patients [13,14] and our results underline once again their impact on mortality. In our analysis, BE was highlighted as a strong protective factor (*p* = 0.008) for death, with a 7% drop of the risk per unitary increase. Even though it is not a predictor of coagulopathy [15], INR has been reported to be a solid risk factor for mortality in trauma patients [16]. In our study this variable, although not significant (*p* = 0.054), was associated with a 38.2% linear growth in the risk of death per unitary increase (OR: 1.382; 95% CI: 0.994–1.922).

All variables identified as predictors of death in our analysis also trigger the activation of massive transfusion (MT) protocols [17]. Nonetheless, we found no differences between survivors and non-survivors in terms of number of blood products transfused. This could be explained assuming that hemorrhagic deaths before 6 or 24 h required MT if the patients died before receiving greater than 10 U of red blood cells (RBCs) in the relevant time interval [18].

E-FAST identified abdominal/pelvic hemorrhage in approximately half of the NCTH patients in our series, and the comparison between survived and deceased patients failed to demonstrate any differences in the rate of positive E-FAST examinations. The presence of hemodynamic instability (massive hemorrhage) does not improve in NCTH patients the possibility of spotting the source of the bleeding [19].

The importance of contrast-enhanced computed tomography (CeCT) in the evaluation of trauma patients is progressively increasing because of its growing diagnostic indications, availability, and the improved speed of CT scanners [20].

Multivariate analysis highlighted CeCT as an independent variable with a protective effect on survival in torso trauma patients. Indeed, patients able to undergo CeCT are hemodynamically stable or responders to fluid challenge/blood products transfusion. These data are supported, in our analysis, by a 73% reduction of risk of death (*p* = 0.029) for patients undergoing CeCT scan.

Recent manuscripts proposed new scores to help predict mortality and morbidity in severe trauma patients, such as the AdHOC score in severely injured patients with major fractures [21]. Our study can be a starting point for identifying new scores useful for planning treatment and minimizing complications in severe NCTIs.

To our knowledge, this study represents one of the largest single-center representations of torso injuries at a Level 1 trauma center in Europe with the review of 777 patient’s data collected in a standardized registry during a nine-year period. Nevertheless, despite the large sample size, the monocentric nature of our study probably represents its main limitation.

Another concern that could be raised is the lack of use of the resuscitative endovascular balloon occlusion of the aorta (REBOA) for unstable torso trauma. The widespread of this hemostatic technique over the years, even in urban areas, is not negligible. However, the comparison of the different therapeutic options goes beyond the aim of our work.

## 5. Conclusions

Torso trauma patients that were hemodynamically unstable in both pre- and in-hospital phases, with impaired GCS and BE have a greater risk of death. Considering the anatomic districts of injury, the involvement of multiple sites of the torso heavily burdens survival outcomes. Physicians must be aware that prompt diagnosis and transport to definitive care facilities (both angio and/or surgical), along with massive transfusion protocols are crucial to give these patients a chance to survive.

## Figures and Tables

**Table 1:** Demographic and trauma-related data. Comparison between survivors and non-survivors.

Variables	Total (*n* = 777)		Survivors (*n* = 650)		Non-Survivors (*n* = 127)		*p*
	Value	%	Value	%	Value	%	
Age (median/IQR)	43	30–59	42	28–57	56	38–56	<0.001 *
Gender (male)	536	69	446	68.6	90	70.9	0.675
Triage code (Red)	335	43.1	317	48.8	18	14.2	<0.001 *
Type of trauma (blunt)	706	90.9	585	90	121	95.3	0.064
Mechanism of trauma							0.014 *
Multivehicle crash	109	14	93	14.3	16	12.6	
Motorcycle crash	224	28.8	198	30.5	226	20.5	
Cyclist	44	5.7	33	5.1	11	8.7	
Pedestrian	120	15.4	98	15.1	22	17.3	
Fall	186	23.9	142	21.8	44	34.6	
Crush	18	2.3	17	2.6	1	0.8	
Gunshot wound	11	1.4	11	1.7	0	0	
Stab wound	55	7.1	49	7.5	6	4.7	
Pre-hospital GCS (median/IQR)	15	11–15	15	14–15	6	3–6	<0.001 *
Pre-hospital SBP (median/IQR)	110	90–130	120	96-130	82	55-82	<0.001 *
Pre-hospital HR (median/IQR)	96	80–115	96	80–110	100	63–95	0.217
Pre-injury ASA-PS > 1	45	5.8	29	4.5	16	12.8	0.001 *
Pre-hospital OTI/LMA							<0.001 *
Not intubated	461	61.6	426	68.5	35	27.8	
Drug assisted	260	34.8	193	31	67	53.2	
Non drug assisted	27	3.6	3	0.5	24	19	
AIS 98’ ≥ 3							
Head	250	32.2	178	27.4	72	56.7	<0.001 *
Chest	600	77.2	492	75.7	108	85	0.021 *
Abdomen	330	42.5	278	42.8	52	40.9	0.769
Pelvis	359	46.2	291	44.8	68	53.5	0.08
Extremities	185	23.8	150	23.1	35	27.6	0.305

* Significant value. IQR: Interquartile Range; GCS: Glasgow Coma Scale; SBP: Systolic Blood Pressure; HR: Heart Rate; ASA-PS: American Society of Anesthesiologists: Physical Status; OTI: Oro-tracheal Intubation; LMA: Laryngeal Mask; AIS 98’: Abbreviated Injury Scale classification 1998 version.

**Table 2:** Patient characteristics, diagnostics, and procedures in the emergency department/operating room. Comparison between survivors and non-survivors.

Variables	Total (*n* = 777)		Survivors (*n* = 650)		Non-Survivors (*n* = 127)		*p*
	Value	%	Value	%	Value	%	
ED GCS (median/IQR)	14	3–15	15	3–15	3	3–3	<0.001 *
ED SBP (mean/SD)	45	19.82	119	28.86	80	46.13	<0.001 *
ED HR (median/IQR)	95	80–110	95	80–110	95	70–95	0.772
Hemodynamic instability	166	21.4	97	14.9	69	54.3	<0.001 *
E-FAST							0.966
Negative	416	53,5	347	53.4	69	54.3	
Positive	320	41.2	268	41.2	52	40.9	
Not performed	40	5.3	35	5.4	6	4.8	
Torso CeCT scan (performed)	679	87.4	591	90.9	88	69.3	<0.001 *
Chest tube							0.003 *
None	434	55.9	369	56.8	65	51.2	
Unilateral	267	34.4	228	35.1	39	30.7	
Bilateral	76	9.8	53	8.2	23	18.1	
DCL	172	22.1	133	20.5	39	30.7	0.014 *
EDT	7	0.9	0	0	7	5.5	<0.001 *
EPP	86	11.1	48	7.4	38	29.9	<0.001*
Angiography/embolization	182	23.4	157	24.2	25	19.7	0.304
BE (median/IQR)	−4	−7/−1	−3	−6/−1	−8	−15/−8	<0.001 *
INR (median/IQR)	1	1–1	1	1–1	2	1–2	<0.001 *
Lactate (median/IQR)	3	2–5	3	2–4	7	4–7	<0.001 *
ISS (median/IQR)	29	21–41	29	20–38	45	35–44	<0.001 *
Probability of death (TRISS)	9	3–35	6	2–20	85	37–85	<0.001 *
Massive transfusion protocol activation	214	27.5	181	27.8	33	26	0.745
PRBCs (median/IQR)	8	5–12	8	5–12	9	5–9	0.84
FFP (median/IQR)	5	4–10	5	4–9	5	2–5	0.409
PP (median/IQR)	1	0–2	1	0–2	1	0–1	0.847

* Significant value. ED: Emergency Department; GCS: Glasgow Coma Scale; IQR: Interquartile Range; SBP: Systolic Blood Pressure; HR: Heart Rate; E-FAST: Extended Focus Assessment Sonography for Trauma; CeCT: Contrast-enhanced Computerized Tomography; DCL: Damage Control Laparotomy; EDT: Emergency Department Thoracotomy; EPP: Extra-peritoneal Pelvic Packing; BE: Base Excess; INR: International Normalized Ratio; ISS: Injury Severity Score; TRISS: Trauma and Injury Severity Score; PRBCs: Packed Red Blood Cells; FFP: Fresh Frozen Plasma; PP: Platelet Pool.

**Table 3:** Multivariate analysis-independent predictors of mortality.

Variables	*p* Univariate	*p* Multivariate	Adjusted OR	95% CI	
				Lower	Upper
Age	<0.001 *	<0.001 *	1.064	1.045	1.083
Pre-hospital GCS	<0.001 *	<0.001 *	0.831	0.771	0.896
Pre-hospital SBP	<0.001 *	0.03 *	0.989	0.980	0.999
Pre-injury ASA-PS (2–3)	<0.001 *				
Pre-hospital OTI /LMA	<0.001 *				
Head AIS ≥ 3	<0.001 *				
Chest AIS ≥ 3	0.023 *				
ED GCS	<0.001 *				
ED SBP	<0.001 *	0.039 *	0.989	0.979	0.999
Torso CeCT scan (performed)	<0.001 *	0.029 *	0.270	0.083	0.874
Chest tube	0.017 *				
DCL	0.012 *				
EDT	N.S.				
EPP	<0.001 *				
BE	<0.001 *	0.008 *	0.931	0.883	0.982
INR	<0.001 *	0.054	1.382	0.994	1.922
Lactate	N.S.				
ISS	<0.001 *	<0.001 *	1.050	1.026	1.075

* Significant value; N.S.: Not Significant; GCS: Glasgow Coma Scale; SBP: Systolic Blood Pressure; HR: Heart Rate; ASA-PS: American Society of Anesthesiologists: Physical Status; OTI: Oro-tracheal Intubation; LMA: Laryngeal Mask; AIS: Abbreviated Injury Scale; ED: Emergency Department; CeCT: Contrast-enhanced Computed Tomography; DCL: Damage Control Laparotomy; EDT: Emergency Department Thoracotomy; EPP: Extra-peritoneal Pelvic Packing; BE: Base Excess; INR: International Normalized Ratio; ISS: Injury Severity Score.
